# Novel Virophages Discovered in a Freshwater Lake in China

**DOI:** 10.3389/fmicb.2016.00005

**Published:** 2016-01-22

**Authors:** Chaowen Gong, Weijia Zhang, Xuewen Zhou, Hongming Wang, Guowei Sun, Jinzhou Xiao, Yingjie Pan, Shuling Yan, Yongjie Wang

**Affiliations:** ^1^College of Food Science and Technology, Shanghai Ocean UniversityShanghai, China; ^2^Laboratory of Quality and Safety Risk Assessment for Aquatic Products on Storage and Preservation, Ministry of AgricultureShanghai, China; ^3^Institute of Biochemistry and Molecular Cell Biology, University of GoettingenGoettingen, Germany

**Keywords:** virophage, genome, metagenomics, freshwater lake, diversity

## Abstract

Virophages are small double-stranded DNA viruses that are parasites of giant DNA viruses that infect unicellular eukaryotes. Here we identify a novel group of virophages, named Dishui Lake virophages (DSLVs) that were discovered in Dishui Lake (DSL): an artificial freshwater lake in Shanghai, China. Based on PCR and metagenomic analysis, the complete genome of DSLV1 was found to be circular and 28,788 base pairs in length, with a G+C content 43.2%, and 28 predicted open reading frames (ORFs). Fifteen of the DSLV1 ORFs have sequence similarity to known virophages. Two DSLV1 ORFs exhibited sequence similarity to that of prasinoviruses (*Phycodnaviridae*) and chloroviruses (*Phycodnaviridae*), respectively, suggesting horizontal gene transfer occurred between these large algal DNA viruses and DSLV1. 46 other virophages-related contigs were also obtained, including six homologous major capsid protein (MCP) gene. Phylogenetic analysis of these MCPs showed that DSLVs are closely related to OLV (Organic Lake virophage) and YSLVs (Yellowstone Lake virophages), especially to YSLV3, except for YSLV7. These results indicate that freshwater ecotopes are the hotbed for discovering novel virophages as well as understanding their diversity and properties.

## Introduction

Virophages are small double stranded DNA (dsDNA) viruses that act as parasites of giant DNA viruses (La Scola et al., 2008; Fischer and Suttle, 2011; Yau et al., 2011; Gaia et al., 2014). Virophages are considered parasitic because they co-infect unicellular eukaryotes along with large DNA viruses, but are dependent on the large DNA virus to replicate which results in the deformation and abortion of the large DNA viruses (La Scola et al., 2008). The first virophage, Sputnik, was isolated in conjunction with the giant DNA virus mamavirus, a relative of mimivirus, from a water-cooling tower in Paris, France (La Scola et al., 2008). Several other virophages were subsequently identified, including Mavirus, a virophage infecting *Cafeteria roenbergensis* virus (CroV), and Zamilon, a close relative of Sputnik, which was isolated with the *Mimiviridae* related virus Mont1 (Fischer and Suttle, 2011; Gaia et al., 2014). In 2015, the viral family of *Lavidaviridae* was proposed for virophages, which currently contains two proposed genera: *Sputnikvirus* and *Mavirus* (Krupovic et al., 2016).

More recently, metagenomic analyses have been utilized to discover diverse and unique virophages from large datasets worldwide. Seven complete genomes of Yellowstone Lake virophages (YSLVs) were assembled from Yellowstone Lake metagenomic datasets (Zhou et al., 2013, 2015). The Organic Lake virophage (OLV) was identified from a hypersaline meromictic lake in Antarctica and was considered as an essential manipulator for the carbon flux and energy cycle through the ecosystem (Yau et al., 2011). A nearly complete genome of Ace Lake Mavirus (ALM) was assembled from Ace Lake metagenomic datasets (Zhou et al., 2013). Early this year, rumen virophages (RVPs), a new group of hybrid virophages of virophage-Polinton, were discovered in a sheep rumen metagenome (Yutin et al., 2015). Interestingly, eukaryotic Polinton transposons appeared to bear viral characteristics (Krupovic et al., 2014) and to be involved in the evolution of eukaryotic virus, transposon, and plasmid (Krupovic and Koonin, 2015). A cryoconite virophage was also assembled from dsDNA viromes from cryoconite hole ecosystems of Svalbard and the Greenland Ice Sheet (Bellas et al., 2015). To date, diverse virophages have been discovered in Europe (La Scola et al., 2008; Desnues et al., 2012; Gaia et al., 2014; Bellas et al., 2015), the Americas (Fischer and Suttle, 2011; Zhou et al., 2013, 2015; Campos et al., 2014), and even Antarctica (Yau et al., 2011; Zhou et al., 2013; Zablocki et al., 2014). However, reports of virophages are lacking in Asia, Africa, and Australia.

In this study, we use PCR and metagenomic analyses to shed light on the diversity and distribution of virophages in freshwater ecosystems in China by examining water samples from lakes and rivers in East China. From these data, a novel group of virophages were identified in Dishui Lake (DSL), Shanghai, China. They are more closely related to YSLVs, especially to YSLV3, than to other known virophages. The DSLVs were consistently detected in DSL and their closely related relatives were found in the neighboring freshwater environments of DSL. The diversity and distribution of the virophages observed in DSL highlight the importance of virophages in China's freshwater ecosystem.

## Materials and methods

### Sampling and DNA extraction

**Figure 1:**
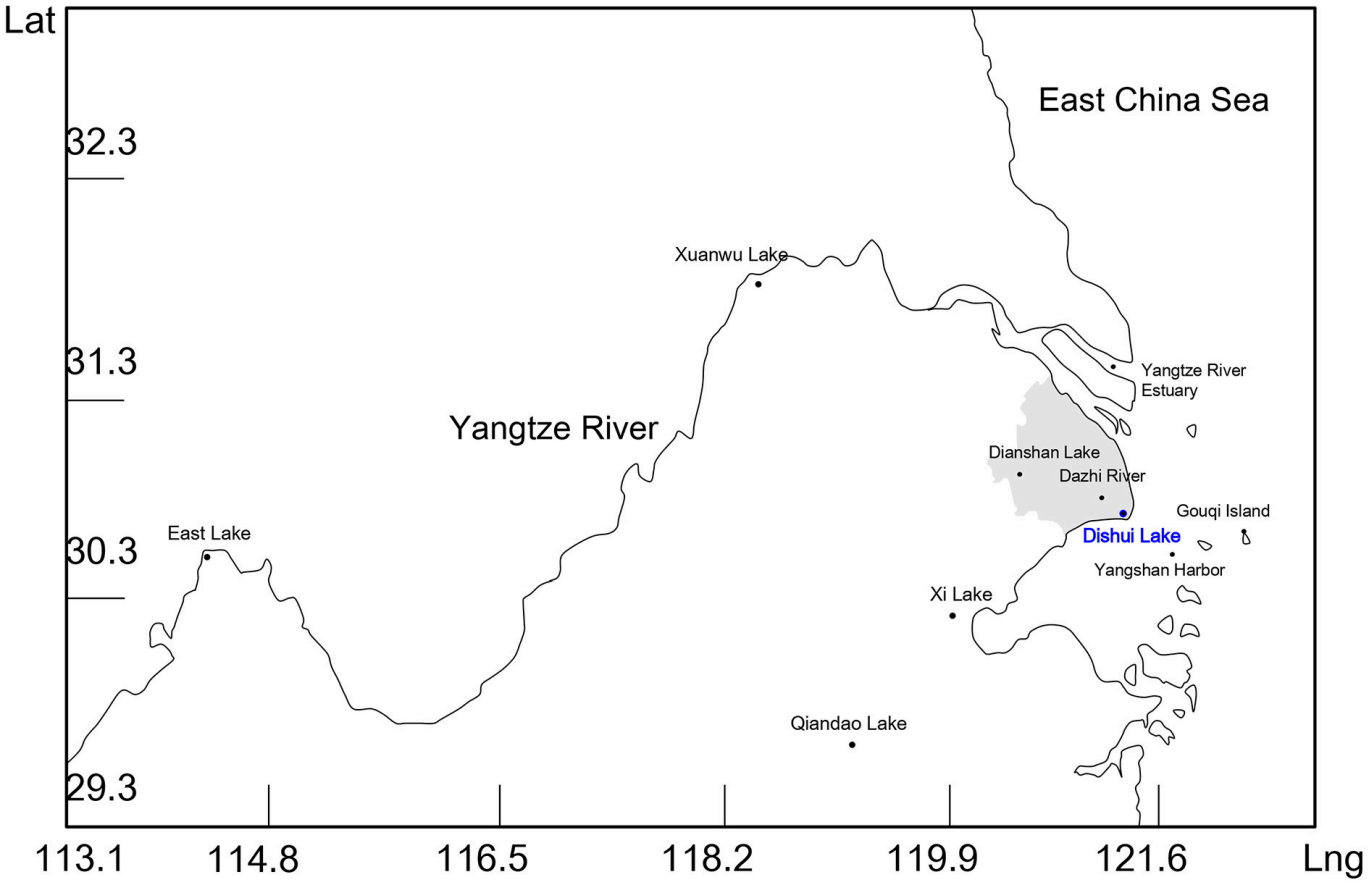
**Map shows the sampling sites in East China**. Sampling sites are indicated with names and dark dots. Dishui Lake is highlighted in blue. Numbers on horizontal and vertical axis represent longitude (Lng) and Latitude (Lat), respectively. The area of Shanghai is shaded in gray.

Surface water samples (depth < 1 m, 500–1000 mL/site) were collected in sterile glass bottles using an electromotion aspiring pump, and kept on ice during delivery to our laboratory. The longitude and latitude of each sampling site were recorded along with sampling time (Table 1, Figure 1). Water samples were immediately filtered through 0.22 μm membrane (GSWP, Merck Millipore) upon receipt. After drying at room temperature, the membrane filters were cut into small pieces, and DNA was extracted from the sample using QIAamp Fast DNA Stool Mini Kit according to the manufacturer's instructions. Final DNA concentrations for each sample were determined using a microplate reader (BioTek) and stored at −20°C before use.

**Table 1:** **The sampling sites and time**.

**Sampling site**	**Longitude (E)**	**Latitude (N)**	**Sampling time**
Dishui Lake	121°55′27.00″	30°53′56.00″	2013/10
Dazhi River	121°46′12.78″	31°00′39.59″	2013/12
Dianshan Lake	120°54′08.76″	31°04′58.55″	2014/09
Yangtze River	121°46′5.16″	31°40′2.76″	2013/06
Yangshan Harbor	122°03′48.9″	30°37′6.42″	2012/11
Gouqi Island	122°45′57.78″	30°42′27.54″	2013/06
Xi Lake	120°09′22.30″	30°15′22.38″	2014/04
Qiandao Lake	118°55′36.84″	29°35′38.90″	2014/04
Xuanwu Lake	118°47′55.49″	32°04′37.53″	2013/12
East Lake	114°25′32.29″	30°33′06.00″	2014/06

### Primers design

Eight pairs of primers were designed using the MCP genes of eight virophages (Ace Lake Mavirus, Mavirus, Sputnik, OLV, YSLV1, 2, 3, and 4; Table 2) and synthesized by Sangon Biotech. Primer specificity was checked *in silico* based on NCBI blast.

**Table 2:** **Eight pairs of virophage MCP genes specific primers designed in this study**.

**Virophage**	**Theoretical length of amplicon (bp)**	**Primer sequence (5**^**′**^ **– 3**^**′**^**)**	
		**Forward**	**Reverse**
Sputnik	534	CTACTACTGCTAGAATTACTGGTGT	ATGCTCCAGAAAGAATACCCTGT
Mavirus	522	ACACCCCCAGAACTCGATAC	ACAACCTAAACCGCGAGACA
ALM	520	TCCGAATGAACCGCCAATAGA	GTTTTGCGTTATGGTTCGGC
OLV	479	AAAGATGGTCCGGCTTCGAG	CTGATGCTAGAGTCGGCACG
YSLV1	426	AGCCGTCGCAATAGTTCCAG	GAAGGTGGTTACGCTACCGA
YSLV2	417	CACCTTTCGTATTTGGCGACC	AGCGGAAGTCGCTTATTCCT
YSLV3	627	CGACCAAGACTTCCAGCCTC	CACAAGTCCCACTGAGTTGC
YSLV4	513	CCATTTCTACCGACCCAGCA	GCACACGAGCGCAAATAAGA

### PCR

PCR reactions (25 μL) contained 0.4 mM forward and reverse primers (Table 2), 12.5 μL *Taq* PCR master mix 2 × (Sangon Biotech), and 20–25 ng of genomic DNA. The PCR cycle conditions are given in Table 3. After agarose gel electrophoresis and ethidium bromide staining, PCR amplicons were visualized with a Gel Doc XR+ system (Bio-Rad). PCR products were purified from the gel using a universal DNA purification kit (Tiangen Biotech), then ligated into the pUCm-T vector (Sangon Biotech). Recombinant plasmids were transformed into TOP10 cells (Tiangen Biotech), which were streaked on agar plates containing 50 μg/mL ampicillin and grown overnight at 37°C. Three positive colonies were selected for Sanger sequencing (Sangon Biotech).

**Table 3:** **Thermal cycling programs of PCR for virophages**.

**Virophage**	**Initial denaturation**	**Denaturation**	**Annealing**	**Extension**	**Final extension**
Sputnik	94°C, 4 min	94°C, 30 s	54°C, 30 s	72°C, 45 s	72°C, 5min
Mavirus					
ALM YSLV1, 2, 3					
OLV			56°C, 30 s		
YSLV4			59°C, 30 s		

### Long-term detection of DSL virophages

Dishui Lake water samples (depth < 1 m, 500–1000 mL) were collected monthly from Oct. 2013 to Sep. 2014. Their DNA extraction, PCR by using the YSLV3 MCP gene specific primers and sequencing were the same as described above.

### Metagenomic sequencing

Up to 6 μg of genomic DNA extracted from Dishui Lake water samples was used to construct two metagenomic libraries that contained 430 bp and 3.0 kb inserts, respectively. Paired-end sequencing (2 × 251 bp) was performed using an Illumina Miseq sequencer (Shanghai Personal Biotechnology).

### QC of metagenomic datasets

The quality of metagenomic datasets was determined using FastQC software (Andrews, 2010). The average quality of five neighboring nucleotides was larger than Q20, with read lengths of more than 50 bp. Following FastQC analysis, the NGS QC Toolkit (Patel and Jain, 2012) was used to filter the reads again, with the default parameters, to ensure high quality reads suitable for assembly.

### The assembly of DSL virophage genomes

Python scripts (Supplementary Material) were modified according to Metavir 2 (Roux et al., 2014) in order to perform sequence alignment for mining virophages related reads in DSL environmental metagenomic data sets. All reads in the DSL metagenomic datasets were firstly aligned onto 12 known virophage genomes using tblastx with *E*-value of 10^−5^. Subsequently, the YSLV3 genome was used as a query to perform tblastx with *E*-value < 10^−10^, which allowed one hit per read, against the DSL metagenomic datasets. Retrieved YSLV3 related reads were assembled de novo with minimum overlap length of 25 bp and minimum overlap identity of 80%. Each of these assembled contigs was used as a reference sequence to which all reads from DSL metagenomic datasets were aligned with minimum overlap length of 25 bp and minimum overlap identity of 90%. The reference assemblies were repeated until the assembled sequences stop extending. All assemblies were performed with the bioinformatics software Geneious Pro (Biomatters).

### PCR verification of DSLV1 genome

Eight pairs of primers (Table 4) were designed to verify regions of the DSLV1 genome where the coverage of reads was low or ambiguous. PCR reactions (25 μL) contained 0.4 mM forward and reverse primers (Table 4), 12.5 μL *Taq* PCR master mix 2 × (Sangon Biotech), and 20–25 ng of genomic DNA. The PCR cycle conditions were as follows: initial denaturation at 94°C for 4 min, followed by 30 cycles of 94°C for 30 s, 54°C (primers 1–4 and 6–8) or 56°C (primer 5) for 30 s, and 72°C for 45 s. Sequences obtained by PCR and sequencing (Sangon Biotech) were then aligned with the assembled genomes to confirm the accuracy of the sequence assembly.

**Table 4:** **Primers for verification of DSLV1 genome**.

**Primer**	**Theoretic length (bp)**	**Primer sequences (5**^**′**^ **– 3**^**′**^**)**	
		**Forward**	**Reverse**
1	417	CTCCTTTTGCGAGGGGAACT	AAGAAACGATGCGAGGTGGT
2	892	CAAATCGCCTAAATGAATGTCCT	CATACCAGTCGCCAGTCCAA
3	605	GGGTAAAACCGCTGGGAGAG	CAGCGGTGTCAAACGCATTA
4	948	TTTCTGTTGCTTACGGGCGA	TGAGTGGAACTTGGAACGCA
5	421	GACCTATCGTCAGGGCAAGG	TAGGAGCGGAAGAAAAGGGG
6	581	ACTTTGGTGAATAGAGCGTTGA	GATAAGGCGTGAGGGTGCTT
7	840	TGCTTATGGCGGACAACCTT	CGGTTTGTGCGTCCAAATCA
8	422	TATACCTGCGTTGGTTGCGT	TAGGTGAGGTAGGTGAGGCA

### Analysis of DSLV1 genome

Geneious Pro software was used to predict virophage open reading frames (ORFs) by stipulating a start codon of ATG and a minimum 150 bp ORF. The predicted ORFs were then searched for homologous genes in NCBI non-redundant (nr) database and a local database containing all known virophage translated ORFs, e.g., Sputnik, Zamilon, Mavirus, OLV, ALM, YLV1-7, using the blastx program. InterProScan 5 and NCBI Conserved Domain Search were used for functional annotation of the predicted ORFs (Marchler-Bauer et al., 2011; Jones et al., 2014).

Whole genome alignment of DSLV1 and YSLV3 was carried out using the Mauve program on the Geneious Pro platform (default parameters; Darling et al., 2004).

### Phylogenetic analysis

The three conserved protein-encoding genes: ATPase, MCP, and Pro from the DSLVs were subjected to phylogenetic analysis. Amino acid sequences were aligned using MUSCLE (Edgar, 2004), and phylogenetic trees were reconstructed using the JTT model with PhyML and 100 iterations (Guindon et al., 2005).

### Detection of DSLVs in neighboring water environments

Water was sampled from Dianshan Lake, Dazhi River, Yangtze River Estuary, Yangshan harbor, and Gouqi island (Table 1, Figure 1). Sampling, DNA extraction and PCR were the same as described above. The YSLV3 MCP gene specific primers were used for screening since they completely matched the target-sequences of DSLV1 MCP gene.

## Results

### PCR detection of virophages

Samples from five different lakes (Xi Lake, Qiandao Lake, Xuanwu Lake, East Lake, and Dishui Lake) in China were analyzed for virophages by using the MCP gene specific primers of eight known virophages (Figure 1, Table 2). As a result, PCR products using these primers were not detected in 4 of the lake water samples: Xi Lake, Qiandao Lake, Xuanwu Lake, and East Lake. However, PCR using the YSLV3 MCP gene specific primers successfully amplified DNA from the Dishui Lake water samples. The length of the PCR products was almost identical to that of the theoretical target sequence of YSLV3 MCP gene (Table 2). The PCR products from Dishui Lake were purified and subjected to cloning and sequencing. The sequences were searched against the NCBI non-redundant database using the blastx algorithm. The best blastx hit was the MCP gene sequence of YSLV6 with an expect value of 8e^−15^ and 33% of identity. When searched against a local dataset containing all translated ORFs of all known virophages, the obtained sequences had the highest sequence similarity with the MCP gene sequence of YSLV3 (89% of identity, *E*-value: 4.3e^−106^).

Additional 12 Dishui Lake water samples collected monthly from Oct. 2013 to Sep. 2014 were virophage positive based on the PCR analysis by using the YSLV3 primers, and their sequences were all the same. It indicated that the virophages existed stably in Dishui Lake.

### Metagenomic sequencing

To ensure both the depth and quality of metagenomic sequencing, six independent runs of Illumina sequencing were performed, which correspondingly yielded six data sets (Table 5). In total, 98,347,716 reads were obtained and subjected to sequence quality control analysis. As a result, 74,429,646 high quality reads were obtained and used for subsequent analysis. The average read length was ~177 bp (Table 5).

**Table 5:** **Information of the DSL metagenomic data sets**.

**Run**	**Raw data**		**QC by the pipeline**		**QC by NGS**	
	**GB**	**Reads**	**GB**	**Reads**	**GB**	**Reads**
1	18.46	35,371,138	6.50	23,505,862	6.43	23,151,200
2	10.82	20,766,464	7.59	17,580,296	7.58	17,507,722
3	3.97	7,974,858	2.53	6,239,280	2.52	6,202,911
4	8.56	17,264,916	5.96	14,037,596	5.95	13,964,065
5	6.97	15,136,922	5.61	12,272,954	5.60	12,216,490
6	0.90	1,833,418	0.59	1,407,000	0.58	1,387,258
Total	49.68	98,347,716	28.78	75,042,988	28.66	74,429,646

### Sequence alignment analysis of metagenomic datasets

**Figure 2:**
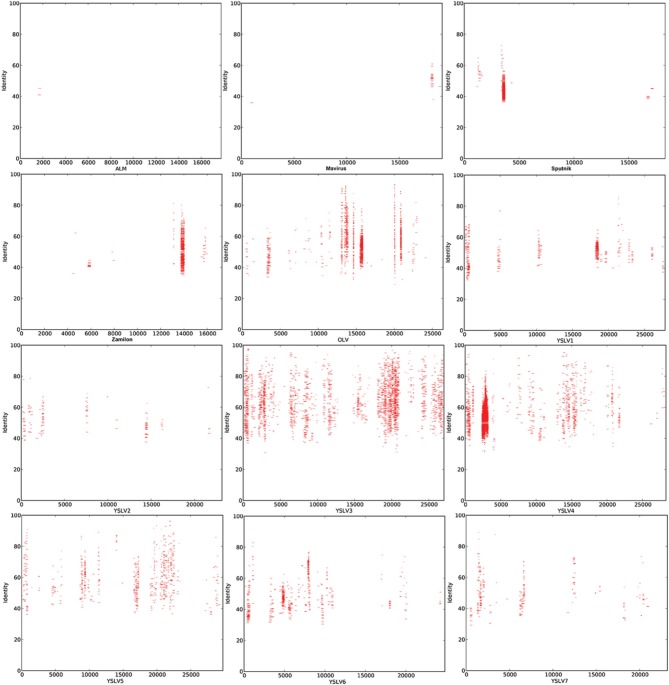
**Sequence alignment analysis of DSL metagenomic data sets**. Red dots represent the recruited reads that shared significant sequence similarity with the virophage genomes. The numbers on the X-axis indicate the position and length (in base pairs) of the virophage genomes. The Y axis shows the percentage of sequence identity shared between the recruited reads and virophage genomic sequences.

To examine the abundance of virophages-related sequences in the DSL metagenomic datasets, sequence alignment analysis was performed by using 12 known virophage genomes. Reads that were similar to OLV and YSLVs (*n* = 3337) were much more abundant than those related to Mavirus, ALM, Sputnik, and Zamilon (*n* = 582) (Figure 2). Notably, YSLV3-related reads were most abundant (*n* = 1199, 30.6% of total reads, *E*-value: 1e^−5^), and 22.4% of reads were similar to the MCP gene of YSLV3 (Figure 2). Accordingly, the YSLV3-related reads were considered for subsequent genomic sequence assembly.

### Assembly of DSLVs

To be more stringent, the YSLV3 genome was used as the query to search against DSL data sets with tblastx and *E*-value of 1e^−10^. 280 YSLV3-related reads retrieved from the DSL metagenomic datasets were assembled *de novo*, resulting in 47 contigs that ranged from 151 to 1560 bp in size (Table 6). All contigs exhibited sequence similarity to that of YSLV3 (Table 6). Therefore, these contigs were used as references for sequence assembly against the DSL metagenomic datasets. As a result, contig1 was the longest assembly of about 28 kb (Table 6) and contained a direct repeat of 189 bp at both ends, indicating the Dishui Lake virophage represented by contig1 contained a circular genome. Eight additional assembled contigs were longer than 5 kb, including four longer than 10 kb.

**Table 6:** **Information of the assembled contigs**.

**Contig**	**Length (bp)**	**Extended length (kb)**	**Best hit in GenBank nr database (*E*-value < 10^−3^)**	**Best hit in virophage ORF database**
1	1560	~28	YSLV6 putative MCP	YSLV3 ORF19
2	1102	~3	YSLV6 putative mCP	YSLV3 ORF18
3	476	~3	YSLV6 putative MCP	YSLV3 ORF19
4	681	~11	OLV OLV4	YSLV3 ORF01
5	732	~7	OLV OLV5	YSLV3 ORF23
6	818	~6		YSLV3 ORF22
7	485	~8	YSLV6 putative mCP	YSLV3 ORF18
8	680	~17	OLV OLV12	YSLV3 ORF06
9	563	~2.6	OLV OLV12	YSLV3 ORF06
10	528	~0.6		YSLV3 ORF13
11	460	~1.6	YSLV6 putative primase-helicase	YSLV3 ORF11
12	532	~3.0	YSLV6 putative MCP	YSLV3 ORF19
13	473	~1.3	Clostridium perfringens DNA adenine methylase	YSLV3 ORF06
14	435	~2.6		YSLV3 ORF19
15	420	~3.5	YSLV6 putative MCP	YSLV3 ORF19
16	391	~1.7	OLV OLV4	YSLV3 ORF01
17	723	~2.2		YSLV3 ORF22
18	639	~13.3	YSLV5 ORF15	YSLV3 ORF09
19	461	~1	YSLV5 ORF11	YSLV3 ORF03
20	445	~3.2	YSLV5 putative cysteine protease	YSLV3 ORF05
21	440	~3.4	YSLV6 putative mCP	YSLV3 ORF18
22	416	~3.6		YSLV3 ORF18
23	375	~3.5	YSLV6 putative MCP	YSLV3 ORF19
24	316	~2.9	YSLV5 putative MCP	YSLV3 ORF19
25	520	~1.2	APMV helicase III/VV D5-type ATPase C-terminus	YSLV3 ORF11
26	390	0.39	YSLV5 ORF15	YSLV3 ORF09
27	302	~1.1	OLV OLV4	YSLV3 ORF01
28	236	0.7		YSLV3 ORF12
29	187	~2.4		YSLV3 ORF13
30	445	0.8	YSLV6 putative packaging ATPase	YSLV3 ORF01
31	441	0.44	YSLV6 putative MCP	YSLV3 ORF19
32	436	~2.7		YSLV3 ORF16
33	434	0.43	OLV OLV5	YSLV3 ORF23
34	434	~15	OLV OLV4	YSLV3 ORF01
35	433	0.43	Hypothetical protein OLV OLV4	YSLV3 ORF01
36	430	0.55	OLV OLV4	YSLV3 ORF01
37	429	0.85	YSLV6 putative MCP	YSLV3 ORF19
38	355	~3.2		YSLV3 ORF13
39	332	0.6	YSLV6 putative MCP	YSLV3 ORF19
40	295	~1.2	Zamilon DNA packaging protein	YSLV3 ORF01
41	292	~5.2	Sputnik V3	YSLV3 ORF01
42	273	0.27	YSLV6 ORF09	YSLV3 ORF10
43	263	0.26		YSLV3 ORF12
44	250	~2.6		YSLV3 ORF19
45	230	~2.1		YSLV3 ORF22
46	210	~1.3	YSLV5 putative primase-helicase	YSLV3 ORF11
47	151	0.78		YSLV3 ORF12

### Verification of DSLV genome

To further verify the ambiguous or low coverage regions of the contig1 as well as to confirm the circular nature of the genome, PCR was performed by using contig1 sequence specific primers and the DSL DNA samples. The obtained PCR products were then cloned and sequenced. After sequence alignment analysis of the PCR amplicons and contig1 genomic sequence, the DSLV genome was determined to be circular, and its length was 28,788 bp with 43.2% of GC content (GenBank accession no. KT894027). We named this virophage DSLV1.

### Genomic and phylogenetic analysis

**Figure 3:**
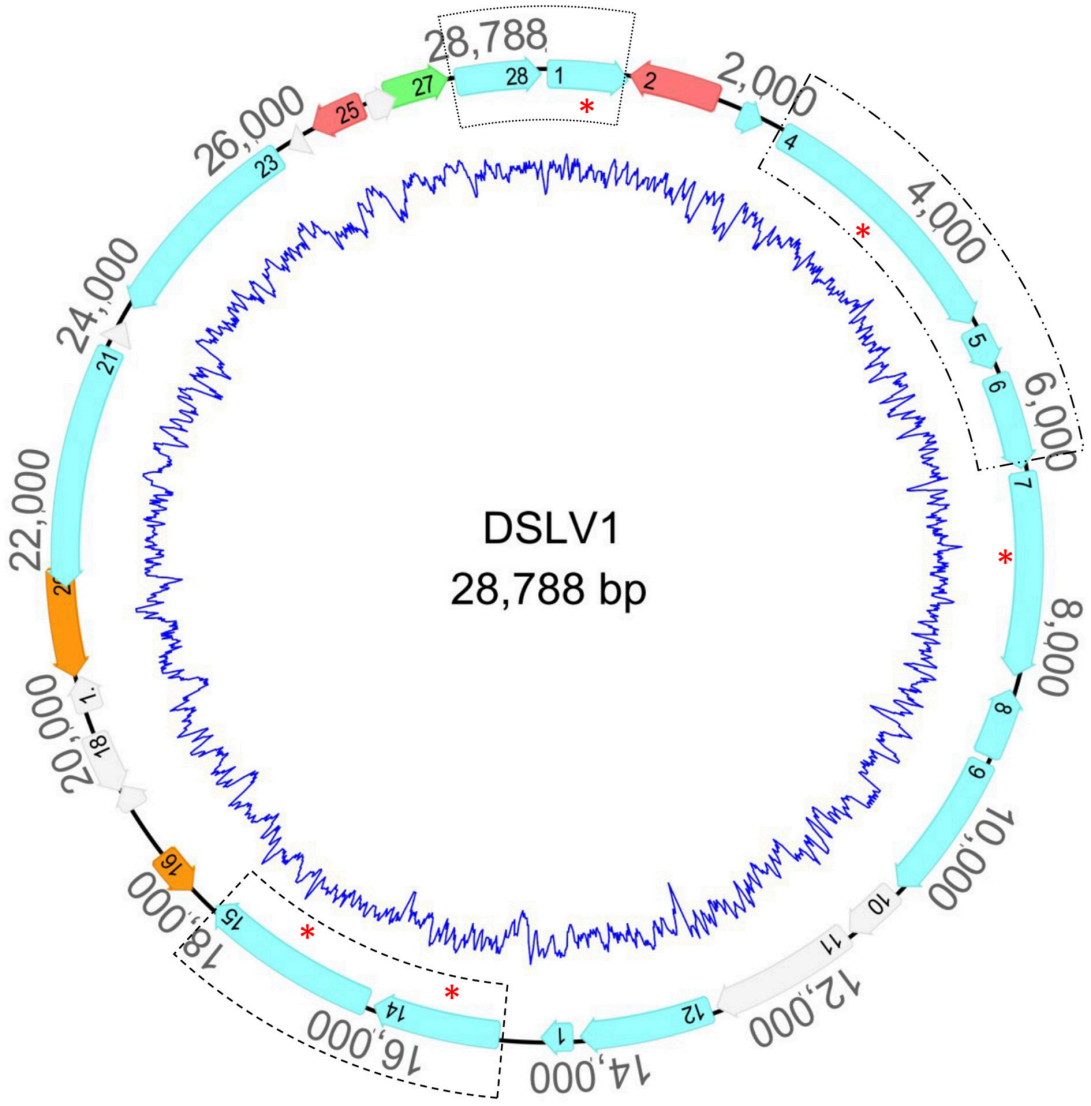
**Circular map of the complete genome of DSLV1**. Homologous genes shared between DSLV1 and YSLV3 are labeled in light blue; DSLV1 and giant algal viruses in red; DSLV1 and cellular organisms in orange; DSLV1 and PgVV (*Phaeocystis globosa* virus 16T virophage) in green. ORFans are marked in light gray. The interior blue line represents %G+C skew throughout the genome. Three gene clusters that have conserved synteny with other virophages are highlighted by the hashed rectangles. Red asterisks indicate the five conserved virophage genes. HEL, ATPase, PRO, MCP, and mCP.

**Figure 4:**
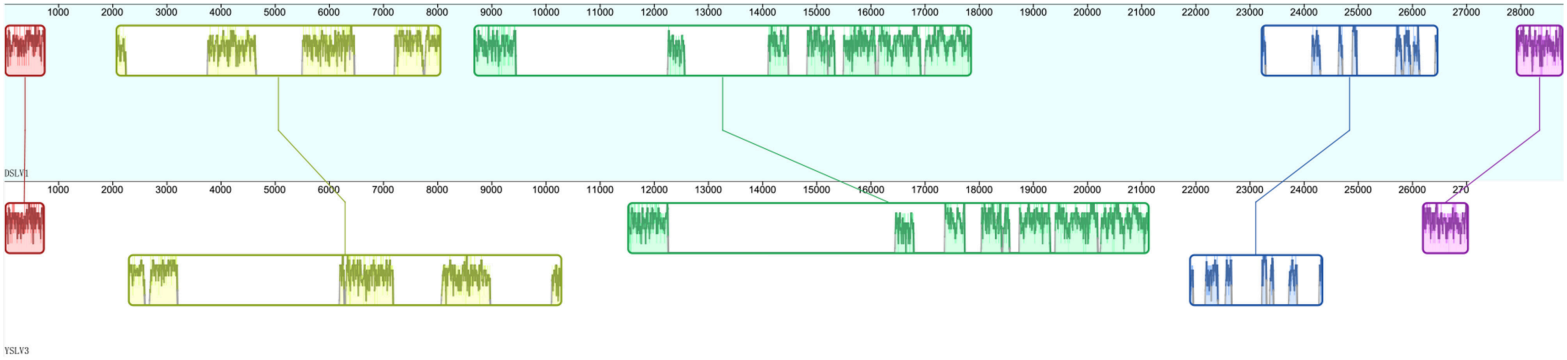
**Whole genome alignment of DSLV1 and YSLV3**. Five conserved genomic regions, shared between DSLV1 and YSLV3, are displayed with rectangles of different sizes and colors.

The DSLV1 genome encodes 28 predicted ORFs, and more than half of the ORFs share higher sequence similarity with YSLV3 (33–70%) than to the other YSLVs, including five conversed virophage genes: putative DNA helicase (HEL), packaging ATPase (ATPase), cysteine protease (PRO), major capsid protein (MCP), and minor capsid protein (mCP) (Figure 3, Tables 7, 8). In addition, whole genome alignments revealed five highly conserved regions that are shared between DSLV1 and YSLV3 (Figure 4). These results suggest that DSLV1 is closely related to YSLV3.

**Table 7:** **Homologous genes present in virophages (modified from Zhou et al., 2013)**.

**Gene product**	**ORF(s) (size in aa) in indicated virophage**												
	**DSLV1**	**YSLV3**	**YSLV1**	**YSLV2**	**YSLV4**	**YSLV5**	**YSLV6**	**YSLV7**	**OLV**	**Sputnik**	**Zamilon**	**ALM**	**Mavirus**
Putative FtsK-HerA family ATPase	01(261)	01(254)	01(256)	01(254)	01(255)	01(313)	01(252)	01(299)	04(256)	03(245)	18(245)	11(334)	15(310)
Putative DNA helicase/primase/polymerase	04(857)	11(865)	04(766)	10(942)	11(880)	31(904)	03(853)		25(777)	13(779)	09(778)	02(553)	01(652)
Putative GIY-YIG endonuclease	03(81)	12(167)	09(225)				16(113)		24(129)	14(114)	08(81)		06(165)
Hypothetical protein	06(311)	09(310)	10(308)	04(344)	14(326)	15(315)	11(296)	08(318)	11(298)		10(168)		
Putative cysteine protein	07(654)	05(172)	23(190)	12(195)	16(191)	19(70)	10(187)	04(175)	07(190)	09(175)	12(188)	10(175)	16(189)
Putative major capsid protein	15(575)	19(578)	25(623)	15(584)	22(617)	21(614)	04(583)	20(585)	09(576)	20(595)	06(609)	08(553)	18(606)
Putative minor capsid protein	14(410)	18(417)	27(477), 26(866)	14(400)	21(394)	20(479)	06(377)	26(383)	08(389)	18(167), 19(218)	05(376)	09(296)	17(303)
Hypothetical protein		03(110)	28(104)	20(101)	26(227)	11(853)	18(98)		02(123)				
Hypothetical protein				02(171)	07(172)								
Hypothetical protein				09(184)								07(143)	
Hypothetical protein				18(204)								17(196)	08(122)
Hypothetical protein	28(276)	23(275)		21(404)	34(325)	06(361)	29(312)		05(290)	21(438)	07(442)		
Hypothetical protein		06(278)			25(421)		07(116), 20(311)	17(134)	12(347)				
Hypothetical protein	05(143)	10(134)			17(139)	07(149)	09(137)						
Hypothetical protein	23(677)	21(554)							10(236)		20(147)	12(262)	14(271)

**Table 8:** **ORFs and their homologs predicted in DSLV1**.

**ORF**	**Position**		**Length**		**Best blastp hit in GenBank nr database and/or local virophage data set**					
	**Start**	**End**	**nt**	**aa**	**ORF, protein encoded**	**Species**	**Accession no**.	***E*-value**	**%aa identity**	**Alignment length in aa (position start-end)**
1	1	786	786	261	Hypothetical protein YSLV3_ORF01	YSLV3		8e^−134^	70	254 (1–254)
2	1649	783	867	288	Hypothetical protein BpV2_168	*Bathycoccus* sp. RCC1105 virus BpV2	ADQ91335	2e^−14^	27	186 (37–222)
3	1876	2121	246	81	Hypothetical protein YSLV3_ORF12	YSLV3		2e^−12^	39	74 (1–74)
4	2328	4901	2574	857	Putative primase-helicase	YSLV3		3e^−169^	37	781 (49–829)
					Hypothetical protein MVEG_12362	*Mortierella verticillata* NRRL 6337	KFH61779.1	1e^−28^	28	285 (475–759)
5	4957	5388	432	143	Hypothetical protein YSLV3_ORF10	YSLV3		1e^−19^	36	129 (15–143)
6	5440	6375	936	311	Hypothetical protein YSLV3_ORF09	YSLV3		2e^−123^	58	307 (4–310)
7	6398	8362	1965	654	Hypothetical protein YSLV3_ORF06	YSLV3		4e^−85^	75	171 (265–435)
					Hypothetical protein YSLV3_ORF05	YSLV3		3e^−62^	55	175 (478–652)
8	9164	8493	672	233	Hypothetical protein YSLV3_ORF13	YSLV3		1e^−69^	50	210 (11–220)
9	9222	10679	1458	495	Hypothetical protein YSLV3_ORF14	YSLV3		7e^−33^	50	152 (1–152)
					Collagen triple helix repeat-containing protein	*Cellulophaga algicola*	WP_013551152.1	4e^−21^	67	90 (265–354)
10	10730	11299	570	189						
11	11340	12761	1422	473						
12	12807	14093	1287	428	Hypothetical protein YSLV3_ORF16	YSLV3		2e^−25^	37	204 (79–282)
								2e^−20^	43	143 (1–143)
13	14150	14464	315	104	Hypothetical protein YSLV3_ORF17	YSLV3		3e^−26^	59	93 (12–104)
14	14858	16090	1233	410	Putative minor capsid protein	YSLV3		1e^−142^	55	408 (2–409)
15	16147	17874	1728	575	Putative major capsid protein	YSLV3		0	68	538 (1–538)
16	18575	18138	438	145	Cysteine desulfurase	*Citreicella* sp. SE45	WP_008883203.1	1.4	26	104 (6–109)
17	19079	19321	243	80						
18	19886	19296	591	196						
19	20100	20450	351	116						
20	21490	20447	1044	347	Serine protease	*Streptococcus sanguinis*	WP_032911859.1	0.010	29	131 (152–282)
21	23639	21303	2337	778	Hypothetical protein YSLV3_ORF22	YSLV3		3e^−40^	37	246 (1–246)
								1e^−34^	33	375 (402–776)
22	23704	23931	228	75						
23	26100	24067	2034	677	Hypothetical protein YSLV3_ORF21	YSLV3		7e^−57^	33	650 (1–650)
								9e^−06^	94	18 (657–674)
24	26396	26205	192	63						
25	27017	26496	522	173	Hypothetical protein NY2A_B677R	*Paramecium bursaria* Chlorella virus NY2A	YP_001497873.1	2e^−31^	45	116 (58–173)
26	27061	27339	279	92						
27	27249	27860	612	203	Hypothetical protein PGVV_00006	*Phaeocystis globosa* virus virophage	AGM15752	8e^−05^	33	85 (112–196)
					Stress response protein NST1	*Beauveria bassiana* D1-5	KGQ04662.1	5e^−05^	30	104 (88–191)
28	27918	28748	831	276	Hypothetical protein YSLV3_ORF23	YSLV3		3e^−123^	63	275 (1–275)

Interestingly, the DSLV1 ORF2 shares 27% amino acid identity with the hypothetical protein BpV2_168 from the giant DNA virus *Bathcoccus* sp. RCC1105 virus BpV2. In addition, DSLV1 ORF25 shared 45% of amino acid identity with the hypothetical protein NY2A_B677R from the *Paramecium bursaria* Chlorella virusNY2A. Such evident genetic link between giant algal viruses and virophages might result from horizontal gene transfer (Table 8).

**Figure 5:**
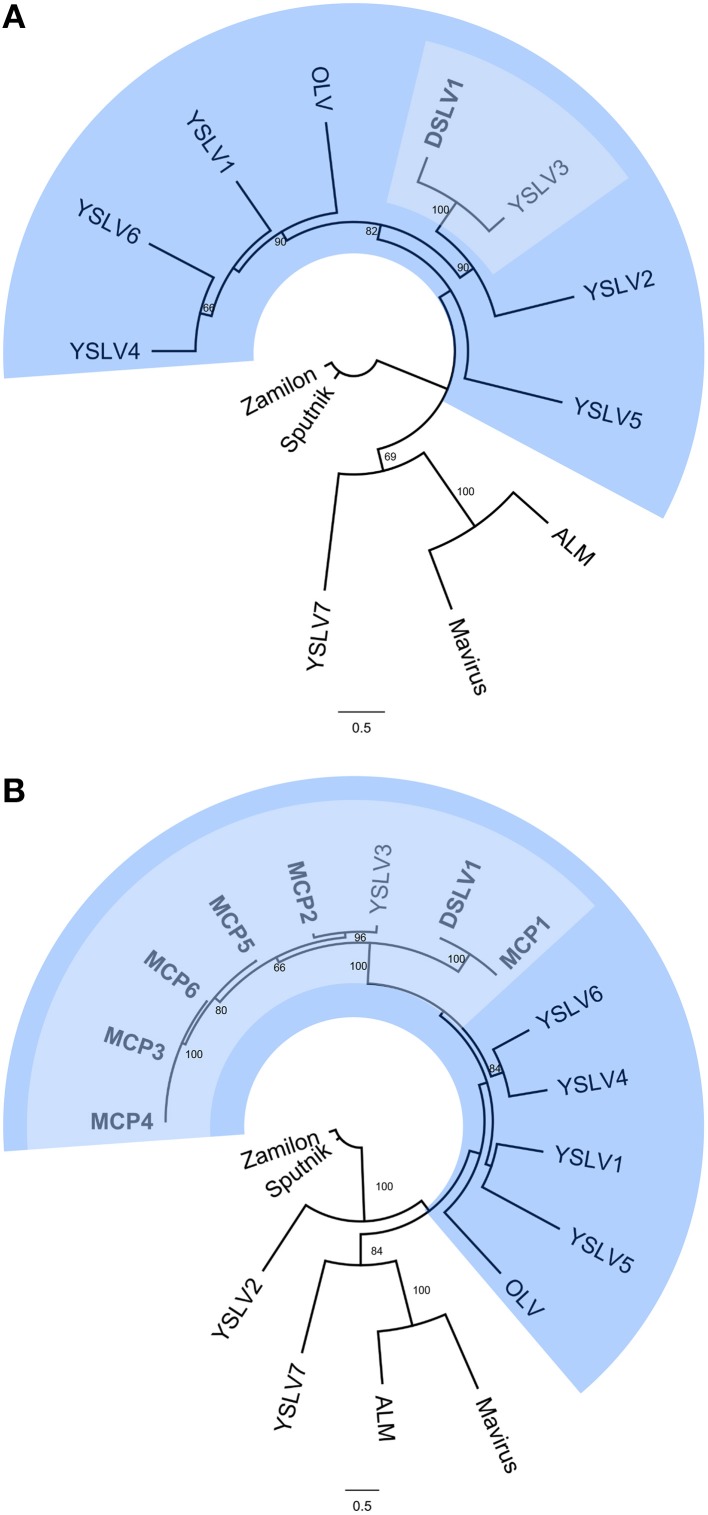
**Phylogenetic trees showing the relationship of DSLVs to other virophages. (A)** A phylogenetic tree was reconstructed based on amino acid sequences of three conserved genes: MCP, Pro, and ATPase to compare DSLV1 to other known virophages. **(B)** Phylogenetic analysis of virophage MCP proteins, including all seven DSLV sequences identified in this study. Bootstrap values are indicated on each branch (100 iterations). DSLVs and YSLV3 are shaded in light blue. DSLVs and the closely related YSLVs and OLV are highlighted in blue. DSLVs are shown in bold. YSLV, Yellowstone Lake virophage; DSLV, Dishui Lake virophage; ALM, Ace Lake Mavirus; OLV, Organic Lake virophage. The accession numbers of the DSLV MCP gene sequences are as follows: KU245924 (MCP1), KU245925 (MCP2), KU245926 (MCP3), KU245927 (MCP4), KU245928 (MCP5), and KU245929 (MCP6).

To better understand the relationship between DSLV1 and previously identified virophages, the concatenated amino acid sequences of three conserved genes of ATPase, PRO, and MCP were used to reconstruct phylogenetic linkages between all the virophages. DSLV1 clustered with YSLV3 (Figure 5A), indicating their closer relationship, as revealed based on the gene content and genome alignment analyses presented above, in comparison to other virophages.

ORFs obtained from the other 46 contigs isolated from Dishui Lake were also analyzed to better understand the diversity of virophages in the Dishui Lake region. In total, we identified six complete MCP genes and six nearly complete MCP genes. All the complete MCP ORFs were used for the phylogenetic tree construction. As shown in Figure 5B, all seven Dishui Lake virophages along with YSLV3 formed a monophyletic group with 100% bootstrap support.

### Distribution of DSLVs in neighboring water environments

To understand whether DSLVs were unique to DSL, water samples were taken from three neighboring freshwater of Dazhi River (inflow of Dishui Lake), Dianshan Lake and Yangtze River Estuary, and two coastal surface sea water of Yangshan harbor and Gouqi island, and screened by PCR for the presence of the DSLVs using the YSLV3 MCP gene specific primers. PCR products were obtained from all three freshwater samples, and when sequenced, these amplicons shared more than 90% of sequence similarity with that of DSLV1. In contrast, DSLVs were not detected in the two sea water samples.

## Discussion

In our previous study, virophages were found to be distributed worldwide, and freshwater environments appeared to contain the highest abundance of virophages (Zhou et al., 2013). In addition, most of the known virophages are distantly related to each other (Bellas et al., 2015; Yutin et al., 2015; Zhou et al., 2015). Therefore, the genetic diversity of virophages and their potential roles in different environments remain enigmatic.

To understand the diversity of virophages in freshwater lakes in China, we performed PCR on samples isolated from five different lakes using virophage MCP genes specific primers. Since the MCP genes of the available virophage genomes are not conserved enough to design degenerate primers, eight pairs of primers were designed individually according to each genome. The presence of virophage in lake surface water was detected in one lake, Dishui Lake, and the presence of these virophages were steadily detectable for a whole year. These results indicate that Dishui Lake virophages existed in the lake over a long time, as opposed to a transient phenomenon, and are worthy of further exploration. In addition, the inability to PCR amplify virophage DNA from the other lakes that surveyed here does not exclude the possibility of far distantly related virophage being present that were not detected using the methods in this study.

To shed more light on the diversity of virophages in Dishui Lake, subsequently, the metagenomic analysis was conducted using the DNA extracts that were virophage positive based on the PCR detection. Initially, the virophage-related MCP gene sequence obtained by PCR was used as a reference sequence for the assembly. However, the sequence assembly terminated after extending to 1.9 kb (data not shown). This suggests that the corresponding virophages were not the most abundant in Dishui Lake. Subsequently, the sequence alignment analysis of the DSL metagenomic data sets revealed that the YSLV3-related virophages were more abundant than virophages related to the other known virophages, such as Sputnik and Mavirus. The seven virophage MCP genes obtained from DSL were phylogenetically grouped with that of YSLV3 as well, which indeed supports the close relationship between DSLVs and YSLV3. This results also suggest that DSLVs and YSLV3 likely infect similar giant viruses and unicellular eukaryotes present in these two lakes, although there are significant differences between DSL and YSL with respect to origin, size, location, geography, physicochemical properties, and human activities (Zhang et al., 2014); http://www.nps.gov/yell/learn/nature/geology.htm). Coincidently, the RNV (Rio Negro virophage), associated with Samba virus, was detected in the Rio Negro River in Brazil (Campos et al., 2014), which shared 100% sequence similarity with the MCP gene of Sputnik that was discovered in a water-cooling tower in Paris, France (La Scola et al., 2008). Taken together, these results suggest that viruses move between different biomes, and their diversity could be high on a local scale but relatively limited globally (Breitbart and Rohwer, 2005; Ignacio-Espinoza et al., 2013).

Interestingly, DSLVs and their close relatives appear to distribute widely throughout the freshwater environments in Shanghai. In contrast, they were not detected in the two neighboring sea water samples, although we did observe some reads in the viral metagenomic data sets derived from Yangshan deep harbor and Gouqi island which were similar to Sputnik (Wang et al., unpublished). Accordingly, these results indicate that the giant virus and the eukaryotic hosts of virophages are likely different in the freshwater and sea water ecotopes.

Both the giant virus and protist hosts of the DSLVs we identified remain unknown. Some virophages share homologous genes with their associated giant viral hosts (Zhang et al., 2015). We identified homologs to both *Bathycoccus* sp. RCC1105 virus BpV2 and *Paramecium bursaria* Chlorella virus NY2A genes in the DSLV1 genome (ORF2 and 25 respectively). Both of these are algae-infecting large dsDNA viruses and respectively belong to the genera of *Prasinovirus* and *Chlorovirus* (King et al., 2011). In a separate study, putative virophage genes as well as giant virus inserts were detected in the nuclear genome of the unicellular alga *Bigelowiella natans* (Blanc et al., 2015). These data suggest that algae possibly serve as the prey of giant viruses as well as their parasitic virophages, and DSLVs may be the parasites of giant viruses that infect algae. Further, work needs to focus on such potential tripartite relationships in China's aquatic ecosystem.

In conclusion, novel virophages were discovered for the first time in an artificial freshwater lake in Shanghai, China, and were found to be widely distributed in the neighboring freshwater bodies. These results further confirm the global distribution and genetic diversity of virophages in freshwater environments.

## Author contributions

Conceived and designed the experiments: SY, YW Performed the experiments: CG, XZ Analyzed the data: CG, WZ, YW Contributed analysis tools: HW, GS, JX, YP Wrote the paper: CG, WZ, YW All authors have read and approved the final manuscript.

### Conflict of interest statement

The authors declare that the research was conducted in the absence of any commercial or financial relationships that could be construed as a potential conflict of interest.
