# Supplementary material for: Novel Virophages Discovered in a Freshwater Lake in China
Source: Front Microbiol. 2016 Jan 22;7:5. doi: 10.3389/fmicb.2016.00005 (PMC4722103; doi:10.3389/fmicb.2016.00005)
Supplement: Supplementary file 1 [file DataSheet1.PDF]

```
#!/usr/bin/python

import matplotlib.pyplot as plt
import os
from Bio import SeqIO

def recuitment_plot(tblastx_result):
    f = open("tblastx_result/%s" %tblastx_result,"r")
    for hang in f.readlines():
        a=hang.strip()
        b=a.split("\t")
        x=[float(b[6]),float(b[7])]
        y=[float(b[2]),float(b[2])]
        plt.plot(x,y,c="red",ls="-",alpha=0.5,animated=False,aa=True,linewidth=1)
    plt.axis([0,length,0,100])
    plt.xlabel("%s" %tblastx_result)
    plt.ylabel("Identity")
    plt.title("Recuitment curve")
    #plt.text(2000,90,"hahaha")
    #plt.grid(True)
    plt.savefig("recuitment_figs/'%s'.pdf" %tblastx_result)
    plt.close()
for i in os.walk("tblastx_result"):
    for k in i[2]:
        for rec in SeqIO.parse("extract_fasta/%s" %k,"fasta"):
            length=len(rec)
        recuitment_plot(k)
```
